# Numbers of cyanovinyl substitutes and their effect on phenothiazine based organic dyes for dye-sensitized solar cells

**DOI:** 10.1039/c7ra13751f

**Published:** 2018-03-09

**Authors:** Yung-Chung Chen, Yuan-Tsung Kuo, Chia-Jung Liang

**Affiliations:** Department of Chemical and Materials Engineering, National Kaohsiung University of Science and Technology 415, Jiangong Rd., Sanmin District Kaohsiung City 80778 Taiwan Republic of China chenyc@kuas.edu.tw; Institute of Chemistry, Academia Sinica No. 128, Sec. 2, Academia Road, Nankang District Taipei 11529 Taiwan

## Abstract

A series of phenothiazine based dyes (OMS1–3), comprising different conjugation lengths and numbers of electron deficient (cyanovinyl) moieties with cyanoacrylic acid as an anchor, have been synthesized. The dyes display broad UV-visible absorption, from 389 nm to 484 nm. The higher molar extinction coefficient and longer absorption peak are achieved as the conjugation length and numbers of electron deficient units increase. The cell performance based on these dyes exhibits efficiencies ranging from 0.68–4.00%, compared to a standard N719-based device (PCE = 7.49%) fabricated under similar conditions. Although the OMS3 dye has two electron deficient units between phenothiazine units, an insignificant electron trapping effect is observed. From the results, the OMS3 based cell exhibits the highest short circuit current (*J*_SC_) at 8.72 mA cm^−2^ and the highest open-circuit voltage (*V*_OC_) at 0.66 V, together with the best cell performance at 4.00%.

## Abstract 2

Phenothiazine based dyes (OMS1–3), comprising different conjugation lengths, numbers of electron deficient (cyanovinyl) moieties have been synthesized. OMS3 dye has two cyanovinyl moieties between phenothiazine core exhibits the best cell performance at 4.00%.

## Graphical abstract

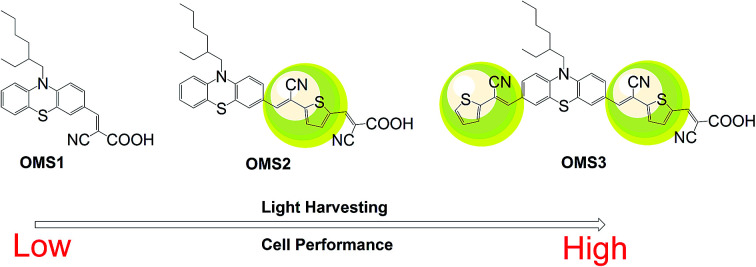

## 1. Introduction

Dye-sensitized solar cells (DSSCs) have become well known candidates for renewable-energy sources since the first report by O’Regan and Grätzel in 1991.^1^ These effective photovoltaic devices are paid more attention because of their inexpensive materials and simple fabrication process. Typical structures of DSSCs comprise a titanium oxide (TiO_2_) semiconductor, electrolyte, counter electrode and a dye sensitizer.^2^ Among these, the most critical factor for a better cell performance is the sensitizer which influences the light harvesting character and electron injection efficiency *etc.* To date, DSSCs based on metal dyes such as Ru complex sensitizers^3^ or zinc-porphyrin sensitizers^4^ have achieved good efficiencies higher than 10% under standard AM 1.5 irradiation. However, these metal dyes are relatively rare, show lower light absorption characteristics and require complicated synthesis and purification processes. Metal-free sensitizers are therefore strong competitors and considered to be an alternative dye sensitizer.^5^ Recently, researchers’ efforts have led to significant cell performance improvements of metal-free dyes. Power conversion efficiencies (PCEs) up to 13% and >14% have been achieved for single dye-based DSSCs and co-dyed systems.^6^ In addition, these metal-free sensitizers possess relatively higher molar extinction coefficients which should render them as good candidates for solid-state, indoor lighting or co-sensitized cells. Traditional metal-free sensitizers used for efficient DSSC devices are generally composed of the structural motif, D–π–A which includes an electron donor (D), an acceptor (A) (the anchor to the nanocrystalline TiO_2_ surface), and a π-conjugated linker (π) between the donor and the acceptor. Sensitizers’ structural modifications influence light absorption (*i.e.* intra-molecular charge transfer transition), electron injection efficiency, dye aggregation and charge recombination properties which are key factors for solar performance.

For instance, a π-conjugated linker incorporates several low-band gap units to form a D–A–π–A configuration due to red-shifted absorption and air stability.^7^ However, the charge trapping effect may occur at some strongly electron deficient moieties^8^ which leads to a less efficient electron injection, hence a lower cell efficiency. Introduction of a cyanovinyl unit is attractive for DSSC application due to the electron withdrawing nature which would increase intramolecular charge transfer and reduce the optical band gap.^9^ In addition, the incorporation of a cyanovinyl unit into the dipolar structure can effectively stabilize the LUMO level.^10^ Although the cyanovinyl unit possesses electron withdrawing characteristics, it shows less charge trapping after appropriate modification of the conjugated spacer *via* quantum chemistry computations with Mulliken charge in the S_1_ electronic transition state.^11^

Phenothiazine-based sensitizers have been widely used in dye-sensitized solar cells.^12^ The phenothiazine structure with electron-rich nitrogen and sulfur atoms possesses stronger electron donor character than other amines, even better than triphenylamine, tetrahydroquinoline, and carbazole. In addition, various structural modifications of the phenothiazine derivatives substituted on C3, C7, N-10 and multi-anchor sensitizers show great potential in tuning the optical properties and solar efficiency.^12^ To the best of our knowledge, phenothiazine-based sensitizers have never been explored by adopting different numbers and positions of electron deficient cyanovinyl units. In this paper, we report the synthesis and characterization of three organic dyes with different conjugation lengths and numbers of electron deficient units at C3 and C7 positions of the phenothiazine core. Moreover, to mitigate dye aggregation and improve the solubility of the dyes, ethyl-hexyl alkyl chains were introduced at the N-10 position of the phenothiazine. The photophysical properties of the compounds and the performance of the DSSCs fabricated with these dyes are discussed.

## 2. Experimental

### 2.1 General information

All solvents used were purified by standard procedures, or purged with nitrogen before use. All the reactions were performed under a nitrogen atmosphere using standard Schlenk techniques. All chromatographic separations were carried out on silica gel (60 M, 230–400 mesh). ^1^H NMR spectra were recorded on a Bruker 400 MHz spectrometer. Mass spectra (FAB) and Fourier-transfer mass spectrometry (FT-MS) were recorded on a Bruker solariX mass spectrometer. Absorption spectra were measured on a Dynamica DB-20 UV-visible spectrophotometer. The photoelectrochemical characterizations of the solar cells were carried out using an Oriel Class A solar simulator (Oriel 91195A, Newport Corp.). Photocurrent–voltage characteristics of the DSSCs were recorded with a potentiostat/galvanostat (CHI650B, CH Instruments, Inc.) at a light intensity of 100 mW cm^−2^ calibrated by an Oriel reference solar cell (Oriel 91150, Newport Corp.). The monochromatic quantum efficiency was recorded through a monochromator (Oriel 74100, Newport Corp.) under short circuit conditions. The intensity of each wavelength was in the range of 1–3 mW cm^−2^. Electrochemical impedance spectra (EIS) were recorded for the DSSC under dark conditions with fixed potential (50 mV) at room temperature. The frequencies explored ranged from 10 mHz to 100 kHz. The standard compound, N719, was purchased from Solaronix, S.A., Switzerland.

### 2.2 Device fabrication

A photoanode TiO_2_ thin film containing a 12 μm-thick transparent layer (solarnix transparent paste) and 6 μm-thick scattering layer (solarnix scattering paste) was used and coated on a FTO glass substrate with a diameter of 0.55 cm. Platinized FTO was produced as a counter electrode by sputtering of Pt. The TiO_2_ thin film was dipped into the THF solution containing 3 × 10^−4^ M dye sensitizer for at least 12 h. After rinsing with THF, the absorbed photoanode adhered with polyester tape of 30 μm in thickness and with a circular aperture of 0.6 cm diameter was placed on top of the counter electrode and tightly clipped together to form a cell. Electrolyte was then injected into the space and the cell was sealed with Torr Seal cement (Varian, MA, USA). The electrolyte was composed of 0.5 M lithium iodide (LiI), 0.05 M iodine (I_2_), and 0.5 M 4-*tert*-butylpyridine which was dissolved in acetonitrile.

### 2.3 Quantum chemistry computation

The computations were performed with Q-Chem 4.0 software.^13^ Geometry optimization of the molecules were performed using the hybrid B3LYP functional and 6-31G* basis set. For each molecule, a number of possible conformations were examined and the one with the lowest energy was used. The same functional was also applied for the calculation of excited states using time-dependent density functional theory (TD-DFT). A number of previous studies exist which employed TD-DFT to characterize excited states with charge-transfer character.^14^ In some cases underestimation of the excitation energies was seen.^13,15^ Therefore, in the present work, we use TD-DFT to visualize the extent of transition moments as well as their charge-transfer characters, and avoid drawing conclusions from the excitation energy.

### 2.4 Synthesis

Compound 1,^16^2, 3, 4,^17^5, and 6 were synthesized as the intermediates. The synthetic procedures and characterization of the new compounds 2, 3, 5, and 6 are described in the following.

#### 2.4.1 Synthesis of compound 2

Compound 1^16^ (1.6975 g, 5 mmol) and thiophene 2-acetonitrile (0.59 mL, 5.5 mmol) were mixed together with 20 mL ethanol as solvent. Then, 10 mol% NaOH solution (1 mL) was added to the flask and stirred at room temperature for 4 h. The mixture was concentrated to remove the ethanol solvent, and the collected organic crude compound was extracted with CH_2_Cl_2_. The crude product was purified by column chromatography using a CH_2_Cl_2_/hexane (3/7) mixture as the eluent. The product was finally obtained as a red oil (1.374 g, 61.8%). ^1^H NMR (400 MHz, CDCl_3_): *δ* 0.85–0.89 (m, 6H), 1.26–1.40 (m, 8H), 1.94–1.98 (m, 1H), 3.76–3.77 (d, 2H, *J* = 6.8 Hz), 6.89 (s, 1H), 6.91 (m, 1H), 6.94–6.98 (m, 1H), 7.04–7.07 (m, 1H), 7.14–7.18 (m, 2H), 7.21 (s, 1H), 7.25–7.27 (dd, 1H, *J* = 5.2, 1.2 Hz), 7.32–7.33 (dd, 1H, *J* = 3.6, 1.2 Hz), 7.55 (d, 1H, *J* = 2.4 Hz), 7.76–7.79 (dd, 1H, *J* = 8.8, 2.4 Hz).

#### 2.4.2 Synthesis of compound 3

A solution of POCl_3_ (0.744 mL, 7.88 mmol) in DMF was added dropwise to a compound 2 (2.9204 g, 6.57 mmol)/DMF complex at 0 °C under a nitrogen atmosphere. The solution was stirred at 0 °C under a nitrogen atmosphere for 30 minutes, then the resulting solution was heated at 80 °C for 12 h to get a red solution. Then, the solution was allowed to cool to room temperature. Aqueous sodium acetate was added to the solution followed by stirring under air for 30 min. Finally, the solution was extracted with CH_2_Cl_2_ and the crude product was purified by column chromatography using a CH_2_Cl_2_ : hexane (1 : 1) mixture as the eluent. The product was finally obtained as a red oil (1.499 g, 48.3%). ^1^H NMR (400 MHz, CDCl_3_): *δ* 0.84–0.89 (m, 6H), 1.25–1.44 (m, 8H), 1.91–1.95 (m, 1H), 3.77–3.79 (d, 2H, *J* = 7.2 Hz), 6.89–6.91 (m, 2H), 6.96–6.710 (t, 1H, *J* = 7.6 Hz), 7.13–7.20 (m, 2H), 7.37 (s, 1H), 7.39–7.40 (d, 1H, *J* = 4 Hz), 7.61 (d, 1H, *J* = 2 Hz), 7.68–7.69 (d, 1H, *J* = 4 Hz), 7.79–7.82 (dd, 1H, *J* = 8.8, 2.4 Hz), 9.86 (s, 1H).

#### 2.4.3 Synthesis of compound 5

Compound 4^17^ (0.7881 g, 2.14 mmol) and thiophene 2-acetonitrile (0.50 mL, 4.71 mmol) were mixed together with 20 mL ethanol as solvent. Then, 10 mol% NaOH solution (0.5 mL) was added to the flask and stirred at room temperature for 4 h. The mixture was concentrated to remove the ethanol solvent, and the collected organic crude compound was extracted with CH_2_Cl_2_. The crude product was purified by column chromatography using a CH_2_Cl_2_ : hexane (4 : 6) mixture as the eluent. The product was finally obtained as a red oil (0.771 g, 62.2%). ^1^H NMR (400 MHz, CDCl_3_): *δ* 0.86–0.89 (m, 6H), 1.25–1.47 (m, 8H), 1.93–1.94 (m, 1H), 3.80–3.81 (d, 2H, *J* = 7.2 Hz), 6.92 (s, 1H), 6.94 (s, 1H), 7.05–7.07 (m, 3H), 7.22 (s, 2H), 7.27–7.29 (dd, 2H, *J* = 5.2, 1.2 Hz), 7.34–7.35 (dd, 2H, *J* = 3.2, 1.2 Hz), 7.53 (d, 2H, *J* = 2.4 Hz), 7.79–7.82 (dd, 1H, *J* = 8.4, 1.6 Hz).

#### 2.4.4 Synthesis of compound 6

A solution of POCl_3_ (0.23 mL, 2.40 mmol) in DMF was added dropwise to a compound 5 (0.5798 g, 1.00 mmol)/DMF complex at 0 °C under a nitrogen atmosphere. The solution was stirred at 0 °C under a nitrogen atmosphere for 30 minutes, then the resulting solution was heated at 80 °C for 12 h to get a red solution. Then, the solution was allowed to cool to room temperature. Aqueous sodium acetate was added to the solution followed by stirred under air for 30 min. Finally, the solution was extracted with CH_2_Cl_2_ and the crude product was purified by column chromatography using a CH_2_Cl_2_ : hexane (2 : 1) mixture as the eluent. The product was finally obtained as a dark red powder (0.170 g, 27.9%). ^1^H NMR (400 MHz, CDCl_3_): *δ* 0.85–0.89 (m, 6H), 1.25–1.42 (m, 8H), 1.91–1.95 (m, 1H), 3.81–3.83 (d, 2H, *J* = 7.6 Hz), 6.94 (d, 1H, *J* = 1.6 Hz), 6.96 (d, 1H, *J* = 1.6 Hz),7.06–7.08 (m, 1H), 7.22 (s, 1H), 7.28–7.30 (m, 1H), 7.35–7.36 (dd, 1H, *J* = 3.6, 1.6 Hz), 7.40 (s, 1H), 7.42–7.43 (d, 1H, *J* = 3.6 Hz), 7.54 (d, 1H, *J* = 2 Hz), 7.62–7.63 (d, 1H, *J* = 2 Hz), 7.71–7.72 (d, 1H, *J* = 4 Hz), 7.81–7.85 (m, 2H), 9.88 (s, 1H).

All the target compounds (OMS1–OMS3) were prepared *via* Knoevenagel condensation by reacting corresponding precursor aldehyde derivatives (1, 3, 6) and cyanoacetic acid in the presence of catalytic amounts of ammonium acetate.

#### 2.4.5 2-Cyano-3-[10-(2-ethyl-hexyl)-10*H*-phenothiazin-3-yl]-acrylic acid (OMS1)

^1^H NMR (400 MHz, CDCl_3_): *δ* 0.84–0.89 (m, 6H), 1.25–1.43 (m, 8H), 1.90–1.96 (m, 1H), 3.79–3.81 (d, 2H, *J* = 7.2 Hz), 6.88 (s, 1H), 6.92 (s, 1H), 6.97–7.01 (t, 1H, *J* = 7.6, 1.2 Hz), 7.12–7.20 (m, 2H), 7.72 (d, 1H, *J* = 2.0 Hz), 7.91–7.94 (dd, 1H, *J* = 8.8, 2.0 Hz), 8.10 (s, 1H). HRMS *m*/*z* [M + Na]^+^ calculated for C_24_H_26_N_2_O_2_S, 429.16072 found: 429.16059.

#### 2.4.6 2-Cyano-3-(5-{1-cyano-2-[10-(2-ethyl-hexyl)-10*H*-phenothiazin-3-yl]-vinyl}-thiophen-2-yl)-acrylic acid (OMS2)

^1^H NMR (400 MHz, CDCl_3_): *δ* 0.86–0.90 (m, 6H), 1.26–1.48 (m, 8H), 1.92–1.95 (m, 1H), 3.78–3.80 (d, 2H, *J* = 7.2 Hz), 6.90–3.93 (m, 2H), 6.96–7.00 (t, 1H, *J* = 7.6, 1.2 Hz), 7.14–7.22 (m, 2H), 7.41–7.42 (m, 2H), 7.70–7.71 (m, 2H), 7.80–7.82 (dd, 1H, *J* = 8.8, 2.0 Hz), 8.32 (s, 1H). HRMS *m*/*z* [M + Na]^+^ calculated for C_31_H_29_N_3_O_2_S_2_, 562.15934 found: 562.15925.

#### 2.4.7 2-Cyano-3-(5-{1-cyano-2-[7-(2-cyano-2-thiophen-2-yl-vinyl)-10-(2-ethyl-hexyl)-10*H*-phenothiazin-3-yl]-vinyl}-thiophen-2-yl)-acrylic acid (OMS3)

^1^H NMR (400 MHz, CDCl_3_): *δ* 0.84–0.89 (m, 6H), 1.24–1.46 (m, 8H), 1.91–1.94 (m, 1H), 3.80–3.82 (d, 2H, *J* = 6.4 Hz), 6.92 (s, 1H), 6.94 (s, 1H), 7.03–7.06 (t, 1H, *J* = 4 Hz), 7.20 (s, 1H), 7.27 (m, 1H), 7.33 (m, 1H), 7.41 (s, 2H), 7.50 (s, 1H), 7.66–7.69 (m, 2H), 7.80–7.82 (d, 2H, *J* = 8.4 Hz), 8.29 (s, 1H). HRMS *m*/*z* [M]^+^ calculated for C_38_H_31_N_4_O_2_S_3_, 671.16146 found: 671.16124.

## 3. Results and discussion

### 3.1 Synthesis

**Scheme 1:**
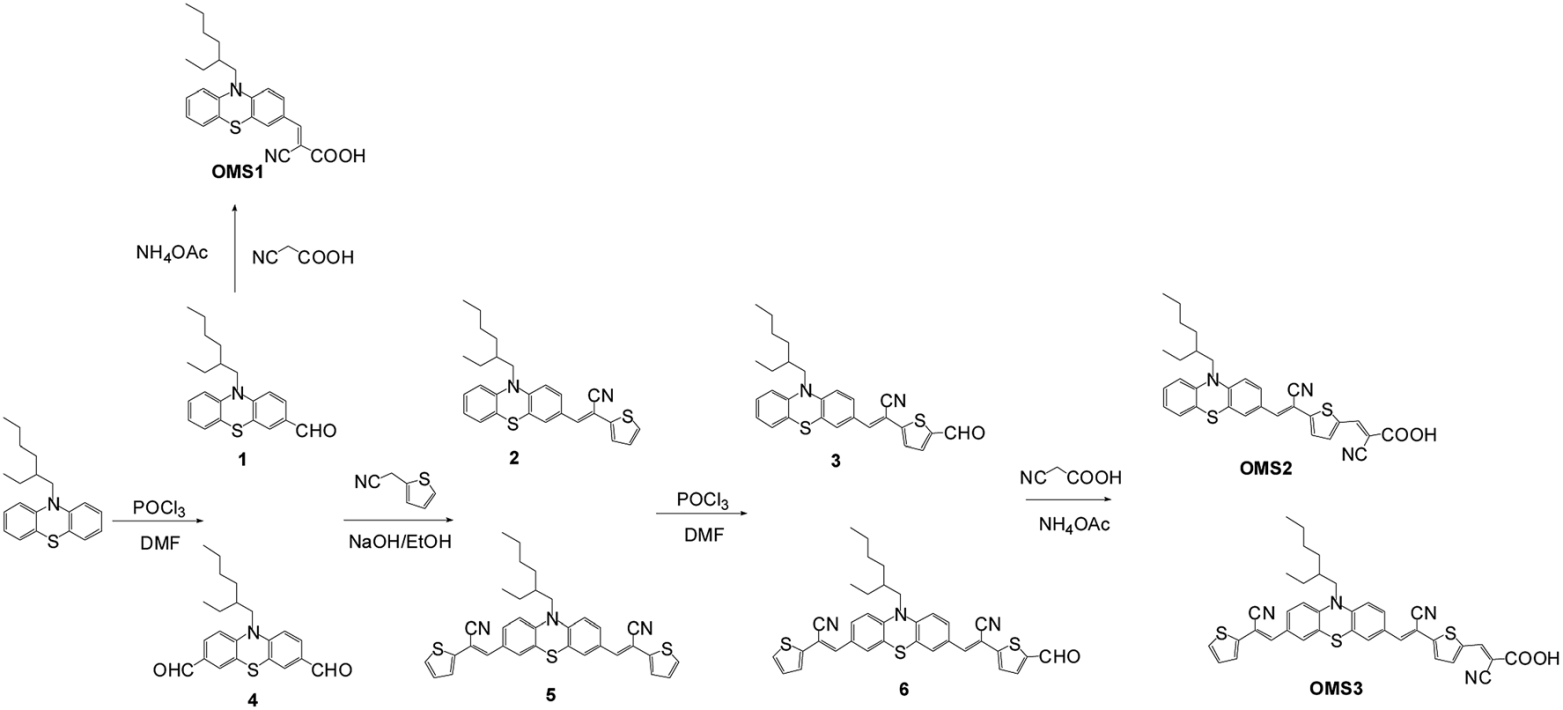
Synthesis of dyes OMS1 to OMS3.

The phenothiazine based organic dyes with a different number of cyanovinyl units (OMS1–OMS3) were synthesized as illustrated in Scheme 1. The intermediates, compounds 1,^16^3, 4,^17^ and 6 with aldehyde functional groups were obtained by Vilsmeier–Haak reaction in the presence of POCl_3_ and dimethylformamide (DMF). The corresponding aldehyde-based compounds were converted into intermediates (2, 5) or target compounds (OMS1–OMS3) through Knoevenagel condensation with 2-thiopheneacetonitrile or cyanoacetic acid, respectively. The final target dyes were obtained as dark red powders, and dissolved in common organic solvents such as THF and CH_2_Cl_2_.

### 3.2 Optical properties

**Fig. 1:**
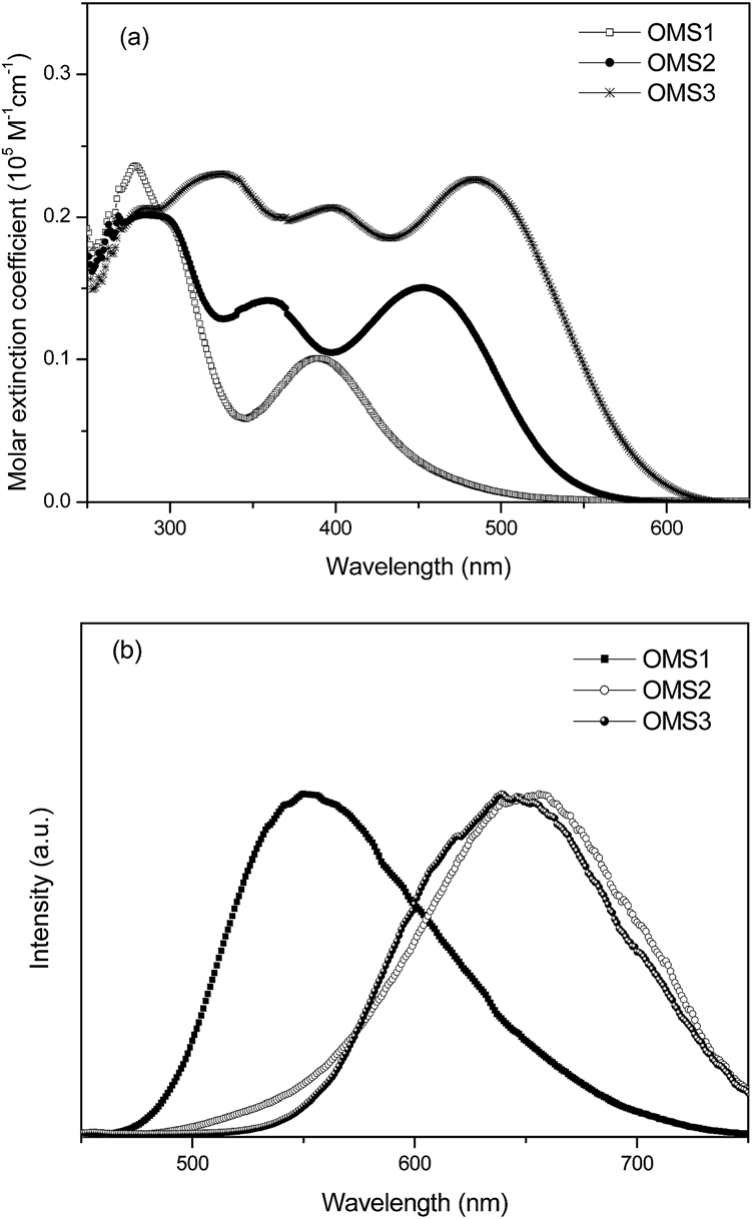
(a) Absorption spectra of the dyes in THF and (b) the emission spectra of the dyes in THF.

The UV-visible absorption spectra of the sensitizers OMS1 to OMS3 in THF solution are shown in Fig. 1(a), and their corresponding data are listed in Table 1. As the electron deficient units increase, the more prominent the absorption bands.^18^ The bands with an absorption maximum below 350 nm are attributed to more localized π–π* transitions. The peak at the longest wavelength for these three dyes is attributed to the intramolecular charge-transfer (ICT) band. Among the three sensitizers, OMS3 exhibits the longest absorption peak due to its longer conjugation length and having more electron deficient units. This can result in a higher degree of electronic delocalization. As expected, OMS3 with the stronger delocalization interaction has the lowest optical band gap of 2.19 eV. These dyes emit in the range of 552 to 653 nm, and follow the trend OMS1 < OMS3 < OMS2 and the emission spectra and the relative results are shown in Fig. 1(b) and Table 1.

**Table 1:** Electro-optical and electrochemical parameters of the OMS dye series

Dye	*λ*_abs_ a (*ε* × 10^4^ M^−1^ cm^−1^) nm	*Λ*_em_ a nm	*E*_1/2_ (ox)^b^ mV	HOMO/LUMO^b^ eV	*E*_0–0_ c eV
OMS1	389 (1.01), 280 (2.36)	552	796 (360)	5.64/3.09	2.55
OMS2	453 (1.50), 360 (1.41), 284 (2.01)	653	551 (97), 769 (155)	5.40/3.11	2.29
OMS3	484 (2.26), 398 (2.07), 332 (2.31)	644	553 (45), 761 (119)	5.40/3.21	2.19

a Recorded in THF solutions at 298 K.

b Recorded in THF solutions. Scan rate, 100 mV s^−1^; electrolyte, ^*n*^Bu_4_NPF_6_; *E*_ox_ = 1/2 (*E*_pa_ + *E*_pc_), Δ*E*_p_ = *E*_pa_ − *E*_pc_ where *E*_pa_ and *E*_pc_ are anodic and cathodic potentials, respectively. Potentials are quoted with reference to the internal ferrocene standard. The HOMO and LUMO energies are calculated using the formula HOMO = 5.1 + (*E*_1/2_ − *E*_Fc_) and LUMO = HOMO − *E*_gap_, where 5.1 refers to energy level of ferrocene *in vacuo*.

c The bandgap, *E*_0–0_, was derived from the intersection of the absorption and emission spectra.

### 3.3 Electrochemical properties

**Fig. 2:**
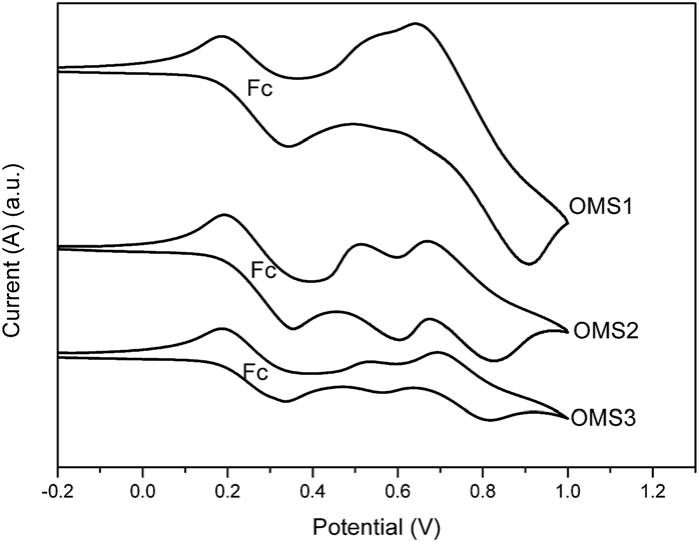
Cyclic voltammograms of OMS1 to OMS3 in deoxygenated THF containing 0.1 M TBAPF_6_ at 25 °C. Ferrocene (Fc) was added as an internal standard. All potentials are in volts *vs.* Ag/AgNO_3_ (0.01 M in MeCN; the scan rate is 100 mV s^−1^).

To judge the feasibility of electron transfer from the excited dye molecule to the conduction band of the TiO_2_ electrode, the redox potentials of these dyes were investigated by cyclic voltammetry (CV) and the data are listed in Table 1. Representative CV spectra are shown in Fig. 2. The redox potentials of these new dyes were measured in THF with 0.1 M tetra-butylammonium hexafluorophosphate. The HOMO levels of OMS1, OMS2 and OMS3 were calculated to be 5.60, 5.40 and 5.40 eV, respectively. The lower oxidation potential of OMS2 compared to OMS1 is attributed to the incorporation of electron deficient units. On the other hand, the introduction of more cyanovinyl electron deficient groups in OMS3 was shown to be insignificant to the oxidation potential compared to OMS2. All of the oxidation potentials of the dyes are more positive than that of I^−^/I_3_^−^ (4.95 eV),^19^ ensuring favorable regeneration of dyes. In addition, the LUMO levels of those dyes were calculated from the HOMO levels and optical band gaps (*E*_0–0_) estimated from the intersection of the absorption and emission spectra. The LUMO levels for the sensitizers OMS1, OMS2 and OMS3 were calculated to be 3.09, 3.11 and 3.21 eV, respectively, which are also lower than the conduction band of TiO_2_ (4.4 eV). This indicates enough driving force for charge injection from the excited sensitizers to the TiO_2_ conduction band.^19^ These results clearly demonstrate that these novel dyes are potentially efficient dyes for DSSCs.

### 3.4 Photovoltaic devices

DSSCs were constructed using these dyes as the sensitizers with the same active area, integrated nanocrystalline anatase TiO_2_ particles, and electrolyte composed of 0.05 M I_2_/0.5 M LiI/0.5 M *tert*-butylpyridine in an acetonitrile solution. The cell performance of the DSSCs was measured under AM 1.5G illumination and is summarized in Table 2.

**Table 2:** DSSC performance parameters of the OMS series dyes

Cell	*V*_OC_ (V)	*J*_SC_ (mA cm^−2^)	FF	*η* (%)
OMS1	0.57	2.09	0.57	0.68
OMS2	0.66	7.18	0.68	3.23
OMS3	0.66	8.72	0.69	4.00
N719	0.77	15.16	0.64	7.49

**Fig. 3:**
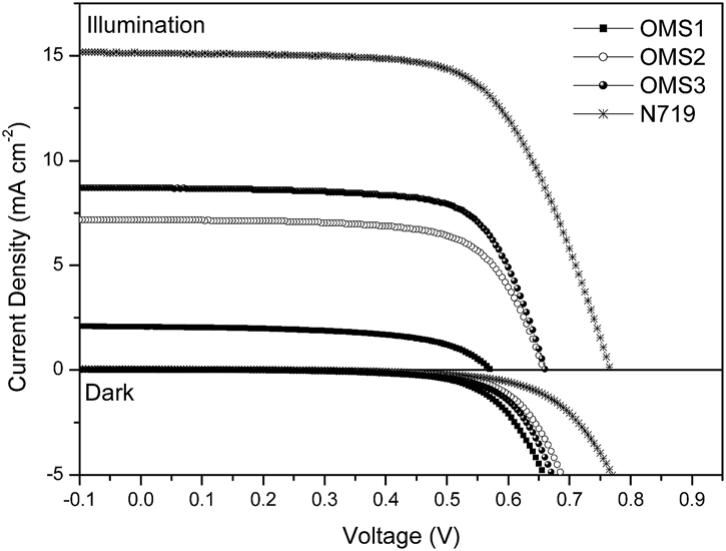
The *J*–*V* curves and dark current of DSSCs based on the dyes.

**Fig. 4:**
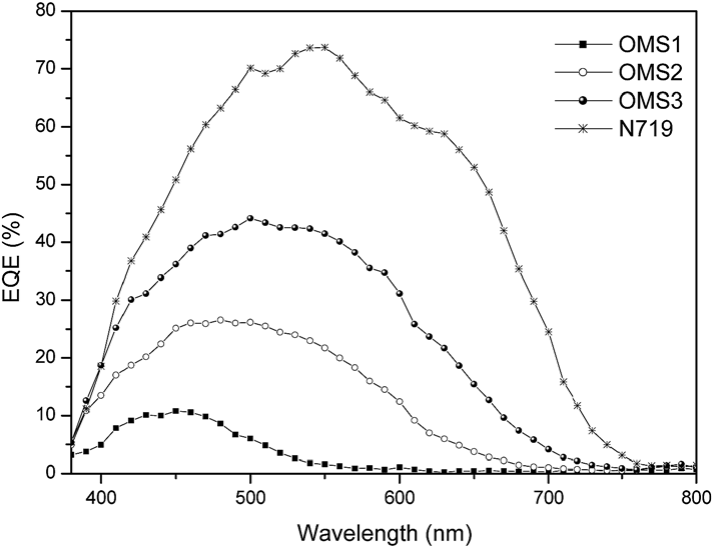
IPCE plots for the DSSCs.

Corresponding photocurrent–voltage (*J*–*V*) curves, dark current and incident monochromatic photo-to-current conversion efficiency (IPCE) plots of the cells are shown in Fig. 3 and 4, respectively. Although the best cell performance (OMS3 = 4.00%) only reached approximately 55% of the standard cell based on N719 (7.49%). The cell performance can be significantly improved by introducing longer conjugation lengths and cyanovinyl moieties to the molecules. The higher *J*_SC_ value of OMS3 compared to OMS1 and OMS2 can be attributed to the following reasons: (1) the absorption spectra of OMS3 appears at a longer wavelength and with better light harvesting than the others; (2) the OMS3 based cell shows boarder and higher IPCE spectra (Fig. 4); (3) the OMS3 suggests a good electron injection efficiency without electron trapping (see theoretical calculations). In addition, the *V*_OC_ data of the DSSCs decreases in the order N719 > OMS2 = OMS3 > OMS1. OMS2 and OMS3 cells with electron deficient units exhibit higher *V*_OC_ values than the congener (OMS1) without the cyanovinyl structure which might be due to the effective suppression of dark current (Fig. 3), although the influence of Fermi-level variation can’t be ruled out.

**Fig. 5:**
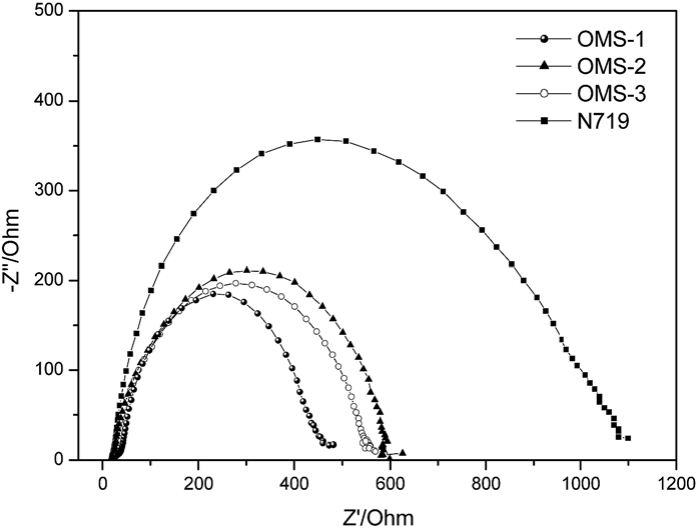
Electrochemical impedance spectra (Nyquist plots) of the DSSCs measured under dark conditions (measured at 50 mV in the dark).

The aforementioned dark current data were also supported by electrochemical impedance spectroscopy (EIS) (Fig. 5). The intermediate frequency represents a charge recombination resistance (*R*_rec_) at the TiO_2_ surface, where the larger *R*_rec_ value implies a smaller dark current. Accordingly, the *R*_rec_ values decrease in the order N719 > OMS2 > OMS3 > OMS1, which is also consistent with the order of decreasing *V*_OC_ values.

### 3.5 Computational studies

**Fig. 6:**
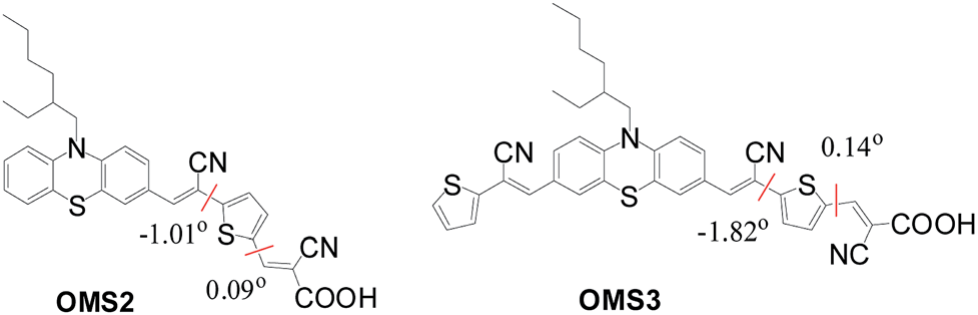
Schematic division and dihedral angles of OMS2 and OMS3 dyes.

The series of dyes OMS1–OMS3 have been modeled by density functional calculations at the B3LYP/6-31G* level of theory. Fig. 6 shows the dihedral angle between the cyanovinyl and thiophene units close to the anchoring group. Insignificant dihedral angles for OMS2 and OMS3 dyes were observed. The smaller dihedral angle of those two dyes leads to better charge transfer and results in red-shifted absorption bands. Hence, the red-shifted and broader absorption of the OMS3 sensitizer may be influenced by extending the cyanovinyl unit far away from the anchors and the smaller dihedral angle.

**Fig. 7:**
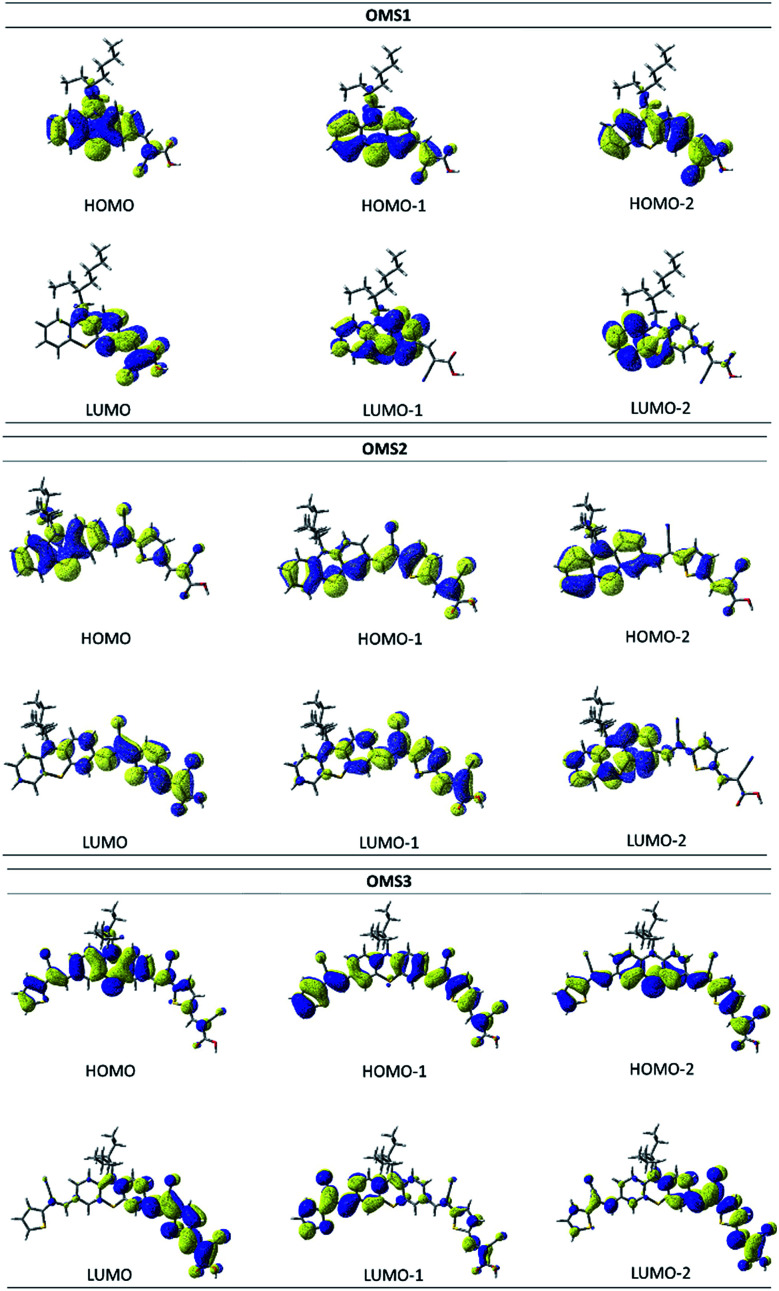
Frontier orbitals of the OMS1 to OMS3 dyes.

Fig. 7 shows the frontier orbitals of the dyes, and the energies, oscillator strengths and the compositions of the lower energy excitations are listed in Table 3. The Mulliken charges of the dyes for the S1 (S_0_ → S_1_), S2 (S_0_ → S_1_) and S3 (S_0_ → S_3_) transitions in different fragments (T′/PTZ/T/Ac, where T′: cyanovinyl far from the anchor; PTZ: phenothiazine; T: cyanovinyl close to the anchor; Ac: 2-cyanoacrylic acid). The HOMOs of the dyes are mainly localized at the phenothiazine core (PTZ), and the LUMOs are mainly localized on the 2-cyano acrylic acid (An). In addition, the LUMOs of the OMS2 and OMS3 dyes extend to localize at the cyanovinyl group (T) close to the anchor. Therefore, the electron deficient moiety of OMS2 and OMS3 dyes is expected to influence the phenothiazine core and lead to a low LUMO energy level.

**Table 3:** Calculated lower-lying transitions of the dyes^a^

Dye	State	Excitation^b^	%	*λ*_cal_, eV	*f* c	*Δ* (Mulliken charge)^d^	
OMS1	S1	H → L	96%	2.63	0.18	PTZ	0.56
						An	−0.56
	S2	H1 → L	92%	3.46	0.20	PTZ	0.54
						An	−0.54
	S3	H → L1	86%	3.97	0.09	PTZ	0.00
						An	0.00
OMS2	S1	H → L	98%	2.07	0.61	PTZ	0.69
						T	−0.40
						An	−0.29
	S2	H1 → L	92%	2.89	0.95	PTZ	0.27
						T	−0.11
						An	−0.16
	S3	H2 → L	7%	3.02	0.01	PTZ	0.54
		H → L1	88%			T	−0.29
						An	−0.25
OMS3	S1	H → L	97%	1.99	0.80	T′	−0.15
						PTZ	0.56
						T	−0.25
						An	−0.08
	S2	H1 → L	21%	2.60	0.33	T′	−0.14
		H → L1	76%			PTZ	0.63
						T	−0.25
						An	−0.14
	S3	H2 → L	36%	2.82	0.11	T′	−0.05
		H1 → L	49%			PTZ	0.32
		H → L1	13%			T	−0.21
						An	−0.02

a Results are based on gas-phase TD-DFT calculation.

b H = HOMO, L = LUMO, H1 = the next highest occupied molecular orbital, or HOMO − 1, H2 = HOMO − 2, L1 = LUMO + 1, L2 = LUMO + 2. In parentheses is the population of a pair of MO excitations.

c Oscillator strength.

d The difference of the Mulliken charge between the ground state and excited state.

In Table 3, the lowest energy transition, S_0_ → S_1_, for all dyes is nearly 100% HOMO → LUMO, therefore it has prominent charge transfer character. Though the OMS3 dye contains two cyanovinyl units, the phenothiazine core still carries most of the positive charge for S1 (0.56) and S2 (0.63) states. Therefore, OMS3 shows less charge trapping as the spacer incorporates suitable cyanovinyl conjugated spacers. The effective charge injection leads to a better current density. In addition, the S1 (S_0_ → S_1_) excitations of the dyes with oscillator strengths (*f*) between 0.18–0.80 (Table 3) are consistent with the measured absorption coefficients. Again, OMS3 exhibits the largest *f* value resulting in an enhancement of the absorption ability.

## 4. Conclusions

In summary, we have synthesized novel phenothiazine dyes consisting of different conjugation lengths, and numbers of electron deficient (cyanovinyl) moieties. The OMS3 dye has a longer conjugation length and electron deficient units between phenothiazine units resulting in better light harvesting and longer absorption bands than the analogous dyes. Furthermore, the more electron deficient units in the OMS3 dye show less impact on the open-circuit values. As a result, the OMS3 based cells exhibit the best performance. Hence, the novel A–D–A–A structure of the OMS3 sensitizer with appropriate molecular design might be useful for improved cell performance.

## Conflicts of interest

There are no conflicts to declare.

## Supplementary Material
